# Evolution of the bacterial nucleosidase PpnN and its relation to the stringent response

**DOI:** 10.15698/mic2019.09.692

**Published:** 2019-07-16

**Authors:** René Lysdal Bærentsen, Ditlev Egeskov Brodersen, Yong Everett Zhang

**Affiliations:** 1 Department of Molecular Biology and Genetics, Aarhus University, DK-8000 Aarhus C, Denmark.; 2 Department of Biology, University of Copenhagen, DK- 2200 Copenhagen N, Denmark.

**Keywords:** ppGpp, YgdH, LOG, DUF4478, DUF3412, PAG

## Abstract

In our recent publication (Zhang *et al.*, 2019), we demonstrate an interesting mode of regulation of purine metabolism unique to Proteobacteria. In this microreview, we would like to reflect on the ideas put forward, with special focus on protein domain architecture of the enzyme involved, its orthologues in plants, and the implications of the differential effects observed between binding of the two alarmone molecules, ppGpp (guanosine 3′,5′-bisdiphosphate) and pppGpp (guanosine-5′-triphosphate-3′-diphosphate). In our previous work, we showed that the *Escherichia coli* nucleotide 5'-monophosphate nucleosidase, PpnN, which is conserved in Proteobacteria, cleaves its preferred substrate, guanosine monophosphate (GMP), at a much higher rate in the presence of both pppGpp and ppGpp (Figure 1A). Structural analysis reveals that binding of pppGpp leads to a conformational change in the protein that exposes its active site, suggesting this is the reason for the observed increase in activity. Finally, point mutation of the alarmone-interacting residues show a defect in binding, resulting in (i) increased basal catalytic activity of PpnN and higher competitive fitness of *E. coli* in an environment with fluctuating nutrient levels, and (ii) increased bacterial sensitivity towards antibiotics. In contrast, complete loss of the *ppnN* gene has the inverse effect, i.e. reduced competitive growth and improved antibiotic tolerance. We used these observations to propose a model in which *E. coli* uses PpnN to balance the need of fitness (fast growth) against tolerance towards antibiotics to improve survival.

## (p)ppGpp IS THE KEY THAT UNLOCKS PpnN

PpnN consists of three domains, a catalytic monophosphate nucleosidase domain (MND) with a Rossmann-like fold, and two Domains of Unknown Function (DUFs) in the N and C termini of the protein, DUF4478 and DUF3412, respectively. The enzyme forms a tetramer via interactions between the N-terminal DUF4478 domain and the neighbouring MND. Moreover, residues from both the N and C-terminal DUF domains come together to form binding pockets for pppGpp between neighbouring monomers. In this way, the DUF domains control access to the active site by structurally occluding it in the *apo* state while exposing it in the pppGpp-bound state. Finally, we show using sequence alignments that the domain structure of PpnN and thus function of the DUF domains is widely conserved across many Proteobacteria. With these new insights in mind, we suggest that DUF4478 and DUF3412 be re-named as *(p)ppGpp-Activated Gating (PAG)* domains 1 and 2 (PAG1 and PAG2), respectively. A search for similar domains in the PFAM database reveals that in the majority of cases, two PAG domains flank a nucleosidase-like domain as observed in PpnN. In a few cases, however, shorter versions of the enzyme are found in which either one PAG domain or the catalytic core plus a PAG domain is missing. Structural analysis suggests that a catalytic core plus a single N-terminal PAG1 domain could be sufficient for oligomerisation (tetramerisation) and thus may support cooperative substrate binding even in the absence of regulation by (p)ppGpp. Alternatively, it is also possible that the truncated proteins represent non-functional PpnN orthologues, but this hypothesis awaits experimental confirmation. Our data show, however, that loss of a functional PpnN enzyme leads to slow growth and render bacteria more tolerant towards antibiotics, a proposed step towards developing resistance. This idea is supported by the observation that several of the truncated *ppnN* genes occur in pathogenic, multi-drug resistant bacteria, some of which have been isolated from patients.

## PpnN SHARES EVOLUTIONARY HISTORY WITH ESSENTIAL PLANT PROTEINS

**Figure 1:**
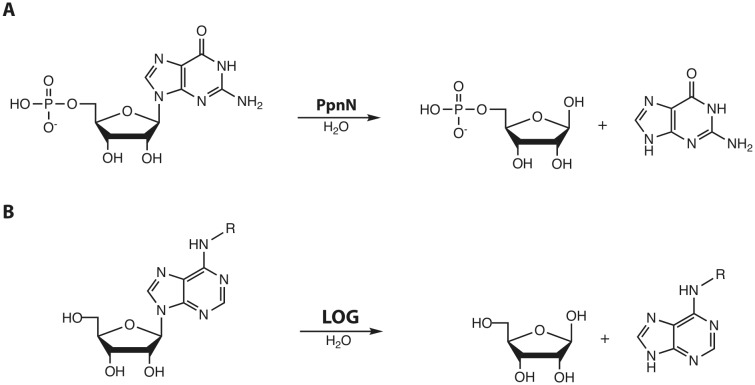
FIGURE 1: Reactions carried out by PpnN and LOG proteins, respectively. **(A)** PpnN hydrolyses 5'-monophosphate nucleotides at the glycosidic bond to yield ribose-5-phosphate and free nucleobase. **(B)** LOG proteins activate cytokinin-type plant hormones by releasing a modified adenine from ribose.

While searching for orthologues of PpnN, we came across a protein family in plants, the so-called LOG ("lonely guy") proteins, which are responsible for the activation of the phytohormone cytokinin through cleavage of the N-glycosidic bond. This reaction is chemically highly similar to the one carried out by PpnN **(Figure 1B)**.

**Figure 2:**
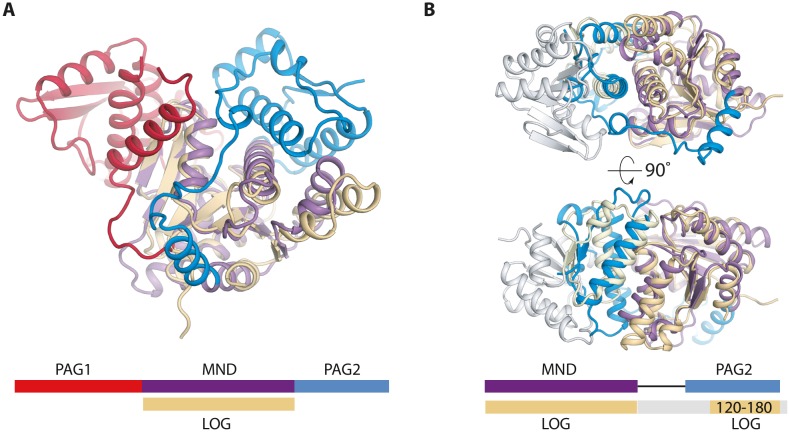
FIGURE 2: Structual comparison of *E. coli* PpnN and a LOG protein (*A. thaliana* lysine decarboxylase-like protein, PDB ID 2A33). **(A)** Comparison between a monomer of PpnN (purple, red, and blue) and LOG (beige) shows that the Rossmann-like fold is shared between the two families. Schematic in same colors below the structure for emphasis. **(B)** Superposition of a LOG dimer (grey and beige) onto the MND and PAG2 domain of PpnN reveals conserved secondary elements between LOG and PAG2 (yellow), more precisely two α-helices and two terminal β-strands. Schematic in same colour below show where the LOG dimer share secondary structure (beige) and the rest is coloured in grey.

There are two available structures of LOG proteins from *Arabidopsis thaliana,* LOG3 and LOG8, both of which form a symmetrical dimer via interactions near the conserved PGGxGTxxE motif, which is predicted to be involved in catalysis. The LOG protein monomer structures are highly homologous to the central MND of PpnN **(Figure 2A)**. Intriguingly, when superimposing one monomer of a LOG dimer onto a PpnN monomer, it becomes evident that there are conserved structural elements between the PAG2 domain and the C-terminal region of the other LOG monomer **(Figure 2B)**. The similarities are found close to the MND of PpnN and cover several structural elements in the C-terminal PAG2 domain that act as a gate to the active site in PpnN. This suggests that the dimerisation of LOG could function to regulate activity in a similar way to the PAG domains in PpnN. It thus appears that there could be an evolutionary connection between PpnN and LOG proteins in which both evolved from a primordial, simpler enzyme consisting only of a single catalytic domain. A gene duplication event could then have led to the presence of one or more PAG domains. Despite the similarities, the two protein families differ and we have found several bacterial PpnN orthologues wrongly annotated as LOG proteins in available databases, which should be revised. We suggest that genes encoding all three domains, PAG1, MND, and PAG2 should be annotated as a *ppnN* orthologue.

## DIFFERENTIAL ALARMONE RESPONSES HINT AT THE SUBTLE COMPLEXITY OF THE STRINGENT RESPONSE

Another interesting aspect not fully discussed in our previous publication is the large shift in activity and cooperativity of PpnN when bound to ppGpp versus pppGpp, especially at the physiologically relevant level of GMP (~24 μM in glucose-fed, exponentially growing *E. coli*). In our study, we tested the catalytic rate of PpnN versus the concentration of the substrate (GMP) in the absence of alarmone or presence of a constant concentration of either ppGpp or pppGpp. When comparing the activity at the physiological substrate concentration, addition of ppGpp only slightly increases the activity of PpnN compared to the situation where no alarmone is present, but surprisingly appears to affect the cooperativity of substrate binding based on the calculated Hill coefficient. Conversely, binding of pppGpp appears to maintain cooperative substrate binding while increasing the activity dramatically. Although a more thorough analysis is required to uncover the kinetics of ppGpp and pppGpp binding to PpnN, as well as how binding affects the subtrate binding, these preliminary data are consistent with what has been found for the *Bacillus subtilis* small alarmone synthetase (SAS), RelQ. In this Gram-positive organism, the alarmone synthesis activity of RelQ is allosterically stimulated by pppGpp, but not ppGpp, suggesting that ppGpp and pppGpp, despite having very similar chemical structures, indeed show differential effects. Furthermore, intracellular concentrations of ppGpp and pppGpp are known to be very dynamic in *E. coli*, ranging from 40 µM during exponential growth, to 810 µM upon transition to stationary phase, and 150 µM in stationary phase, respectively. Additionally, a preferential production of either ppGpp or pppGpp is observed in various strains when exposed to different types of stress. In *E. coli*, the enzyme GppA is known to quickly convert pppGpp to ppGpp. Finally, other ppGpp analogues such as pGpp, ppGp, or pGp have recently been discovered by both us and other groups as well. Whether and how these molecules affect the activity of PpnN is currently unknown. Moreover, we know little about how the activity of PpnN is regulated temporally during bacterial adaptation to stress. Similarly, it is unclear how the regulation of PpnN in particular, and other (p)ppGpp targets in general, contribute to bacterial fitness. These questions remain important to answer, and are currently under study in our laboratories.

## CONCLUSION

Recently published data on the bacterial nucleosidase PpnN has helped clarifying how *E. coli*, and likely most other Proteobacteria, regulate their nucleotide metabolism in response to the stringent response. Environmental stress initiates the stringent response which is marked by a rapid accumulation of the alarmones pppGpp and ppGpp. Allosteric binding of the alarmones increases the activity of PpnN, which downregulates the purine biosynthesis, ultimately reducing the growth rate of the bacteria, but concomitantly increasing tolerance towards stress. Moreover, this work also led us to uncover the molecular function of two previously uncharacterised (DUF) protein domains. Some LOG proteins, and some PpnN orthologs, have been wrongly annotated as lysine decarboxylases. Studies have already shown that the LOG proteins rather function as nucleosidases and not lysine decarboxylases, and this argument can be extended to PpnN. With the new data in hand we propose a domain nomenclature in which PpnN-type enzymes consist of the newly identified PAG1 (formerly DUF4478), MND (based on its Monophosphate Nucleosidase Domain activity), and PAG2 (formerly DUF3412) domains.

Constant improvements in the limits and methods for detecting alarmones, both *in vivo* and *in vitro* means that we can start to answer more complex questions as above with regards to the stringent response. We already have data that shows that PpnN has a different response to two very similar molecules, ppGpp and pppGpp, indicating that the regulation is more complex than an ON/OFF switch. Testing the activity of proteins at alarmone pool levels resembling natural growth states could help us gain a greater insight into how bacteria regulate their growth, both with and without stress.

